# Unveiling the link between ACR TI-RADS grading and Bethesda score of thyroid nodules in diabetic patients: A comprehensive analysis

**DOI:** 10.17305/bb.2024.10670

**Published:** 2024-12-01

**Authors:** Ying Wang, Xi Chen, Yu Chen, Fei Xie, Zhuoyan Wang, Runyue Mao, Ligang Wang

**Affiliations:** 1 Center for General Practice Medicine, General Practice and Health Management Center, Zhejiang Provincial People’s Hospital (Affiliated People’s Hospital), Hangzhou Medical College, Hangzhou, Zhejiang, China; 2 Cancer Center, Department of Ultrasound Medicine, Zhejiang Provincial People’s Hospital, (Affiliated People’s Hospital), Hangzhou Medical College, Hangzhou, Zhejiang, China

**Keywords:** Type 2 diabetes mellitus (T2DM), thyroid nodules (TNs), American College of Radiology Thyroid Imaging Reporting and Data System (ACR TI-RADS) grading, Bethesda score, logistic regression analysis

## Abstract

This study aimed to explore the factors influencing thyroid nodules (TNs) in individuals with type 2 diabetes mellitus (T2DM) and evaluate the consistency between different American College of Radiology Thyroid Imaging Reporting and Data System (ACR TI-RADS) grades and Bethesda scores. A total of 642 T2DM patients were divided into the TN group (245) and the control group (397) based on the presence or absence of TNs. TN patients were further categorized into ACR TI-RADS classification (TR) 1–4 subgroups and TR5 subgroups. Diabetes-related clinical and biochemical parameters were collected, and differences were analyzed using univariate analysis. Logistic regression analysis was utilized to pinpoint independent influencing factors for TN occurrence and different TN classifications. Consequently, age, body mass index (BMI), fasting plasma glucose level (FBGL), low-density lipoprotein cholesterol (LDL-C), diabetic progression, and family history of TNs emerged as independent risk factors for TN development in T2DM patients. Additionally, glycosylated hemoglobin (HbA1c), nodule diameter, and family history of TNs were identified as independent risk factors for TR5 TN development in T2DM patients. All TR1–2 nodules had a Bethesda score of 2 and all showed benign pathological findings. In 97.10% of cases (67/69), nodules classified as TR3 exhibited a Bethesda score of 2, with all pathological results indicating benign findings, aligning with the Bethesda score. In addition, the concordance between TR4 nodules and Bethesda score was only 78.57% (88/112). In conclusion, TNs and their malignancy in T2DM patients are significantly linked to blood glucose and lipid metabolism indices. TR3 classification in T2DM patients poses a low malignancy risk, suggesting caution when conducting fine needle aspiration cytology (FNAC) testing.

## Introduction

The thyroid gland stands out as a pivotal player in the human endocrine system [1]. The thyroid gland is mainly regulated by the hypothalamus–pituitary gland, and its synthesized and secreted thyroid hormones play a vital role in human growth, development, and the regulation of glucose and lipid metabolism, among other functions [1]. Thyroid nodules (TNs), common maladies of the endocrine system, denote isolated anomalies characterized by localized abnormal growth of thyroid cells distinctly separated from surrounding tissue [2]. The occurrence and development of TNs are mainly caused by genetic predisposition, environmental factors, abnormal iodine intake, dietary habits, and other influences [3]. In the absence of prior imaging techniques, TNs can only be detected by palpation by experienced clinicians with an incidence between 4% and 7%, with a considerable rate of underdiagnosis [4]. Typically, TNs have no obvious clinical manifestations, appearing solely as palpable masses moving within the anterior cervical region upon swallowing [5]. However, as nodules expand, they may cause neck enlargement and compression symptoms like dysphagia and dyspnea [5]. With the development of imaging techniques, the diagnostic efficacy of TNs has markedly improved. The detection rate via color ultrasonography ranges from 20% to 76%, with malignancies accounting for 7% to 15% [6]. As per 2022 statistics from the Chinese Cancer Center, thyroid cancer ranked seventh among various cancers, and the prevalence was significantly increased compared with that of five years ago [7]. Therefore, regular ultrasonography for TN detection and preventative measures against malignant TNs are imperative.

Epidemiological investigations have unveiled a close association between type 2 diabetes mellitus (T2DM) and TN occurrence [8]. Studies have shown that the risk of TNs in patients with T2DM is 1.78-fold higher than that in healthy individuals [9]. Nonetheless, the underlying pathogenesis of T2DM and TNs remains elusive. Presently, insulin resistance (IR) is a widely accepted mechanism by many researchers. Clinical studies reveal significantly higher IR levels in TN patients compared to non-TN patients, with a notable correlation between IR and TNs [10]. Further studies indicate that the size of TNs increases with elevated IR index (HOMA-IR) in patients with T2DM [11]. Moreover, findings from Blanc et al. [12] suggest that higher glycosylated hemoglobin A1c (HbA1c) levels may serve as a risk factor for TN formation and tissue growth in elderly patients with metabolic syndrome, correlating with altered thyroid morphology. Given the intricate relationship between diabetes mellitus and TNs, the influencing factors for TNs in the diabetic population are unclear.

Presently, the optimal utilization of ultrasound to discern clinically significant thyroid cancer remains a focal point of recent research. The American College of Radiology Thyroid Imaging Reporting and Data System (ACR TI-RADS), proposed by the American College of Radiology (ACR), represents the latest risk stratification criteria. ACR TI-RADS primarily assigns relative scores based on TN composition, echogenicity, morphology, margins, and echogenic foci, thereby enhancing the diagnostic precision of TNs [13]. Additionally, Fine Needle Aspiration Cytology (FNAC) serves as the primary diagnostic tool to differentiate benign from malignant TNs, with cytopathological diagnoses classified by the Bethesda Reporting System. However, literature reports indicate that approximately 20% of FNAC results are inconclusive due to unsatisfactory specimens [14]. Currently, the gold standard for diagnosing benign and malignant TNs remains pathological biopsy following surgical resection. Moreover, FNAC represents an invasive procedure, and given that most nodules are benign and do not necessitate FNAC, unnecessary procedures should be minimized. To address this, we analyzed factors influencing TNs across different ACR TI-RADS risk stratifications. Concurrently, this study aimed to assess the consistency between different ACR TI-RADS classification (TR) nodules and Bethesda scores in diabetic patients, thereby facilitating more accurate diagnosis of benign and malignant TNs and reducing unnecessary FNAC procedures.

## Materials and methods

### Study subjects

According to the inclusion and exclusion criteria, 642 patients with T2DM, aged 29–81 years, were selected from the General Practice Health Management Center of Zhejiang Provincial People’s Hospital from June 2020 to June 2023. The patients’ diagnoses of T2DM were consistent with the Chinese guidelines [15]. Inclusion criteria included: (1) meeting the diagnostic criteria of T2DM; (2) undergoing thyroid color ultrasonography; (3) completing FNAC for nodules ranked 3–5 in the ACR TI-RADS system, and obtaining cytopathological diagnosis and classification by Bethesda reporting system; (4) all nodules with FNAC were found to have histopathological results; and (5) patients were required to sign informed consent. Exclusion criteria included: (1) patients with a history of thyroid surgery; (2) patients with thyroid metastasis; (3) patients with a history of neck radiation; (4) patients with other malignant tumors; and (5) patients with severe heart, liver, and kidney dysfunction.

### Clinical data

Demographic and medical information, including age, sex, duration of diabetes, and family history of TNs, was collected for all participants. Height and weight were also measured to calculate body mass index (BMI). Blood samples were taken in the morning after a 10-h fast to determine levels of fasting plasma glucose (FBGL, mmol/L), fasting insulin (FINS, mIU/L), glycosylated hemoglobin (HbA1c, %), triglycerides (TG, mmol/L), total cholesterol (TC, mmol/L), high-density lipoprotein cholesterol (HDL-C, mmol/L), and low-density lipoprotein cholesterol (LDL-C, mmol/L). IR index (HOMA-IR) was calculated from FBGL and FINS. HOMA-IR ═ (FBGL×FINS)/22.5 [16].

### Thyroid examination

All patients underwent thyroid color ultrasonography using a 7.5 MHz probe and the HS-2000 color Doppler ultrasound machine (Honda Electronics Co., Ltd.). The procedure was performed while the patients were in a supine position with their anterior cervical region fully visible. All ultrasound examinations were performed by a senior physician with more than five years of experience in ultrasound diagnosis of the thyroid gland. If a TN was detected, its size, boundary, location, echo, morphology, and presence of calcification were noted. The nodules were then classified using the ACR TI-RADS system [13]. ACR TI-RADS risk stratification interpretations were evaluated individually by two experienced senior physicians in a double-blind manner. In cases of disagreement, a third senior physician at the rank of deputy director or higher was consulted. The final conclusion was reached through joint consultation. All TNs were measured in a three-dimensional manner and the largest diameter was recorded to assess TN size regardless of the number of nodules [17].

### Thyroid fine needle aspiration cytology

The patient was instructed to take a supine position and place his shoulder and neck high so that it was in an extended position to fully expose the anterior cervical region. The anterior cervical area is routinely disinfected. After local anesthesia, a 22 G needle was punctured into the TN under ultrasound guidance and the needle core was removed. Remove a few tumor cells by suction and collect cell debris by rapid lifting and inserting back and forth under negative pressure. After the operation, the puncture point was pressed for hemostasis. According to the classification criteria of thyroid cytopathology, the Bethesda score was divided into six categories: 1 as not diagnostic value or dissatisfactory, 2 as benign, 3 as atypical hyperplasia of unclear significance or follicular hyperplasia of uncertain significance, 4 as a follicular tumor or suspicious follicular tumor, 5 as suspicious malignant, and 6 as malignant [18]. Cytological aspirate samples with a Bethesda score of 1, indicating no value or unsatisfactory results, were not included in the study. Samples with a score of 3 or 4 were not included because they could not be confidently classified as either benign or malignant.

**Table 1:** Univariate analysis of thyroid nodules in diabetic patients

**Variable**		**Thyroid nodule (*n* ═ 245)**	**Control (*n* ═ 397)**	**t/Z/X^2^**	***P***
Age (years)		56.00 (51.00, 61.00)	47.00 (43.00, 52.00)	5.344	<0.001
Sex	Male	118	171	1.586	0.208
	Female	127	226		
BMI (kg/m^2^)		26.42 ± 2.53	24.96 ± 2.83	6.771	<0.001
FBGL (mmol/L)		9.24 (8.27, 10.07)	8.31 (7.60, 9.30)	3.468	<0.001
FINS (mIU/L)		17.70 (12.66, 20.38)	14.48 (11.73, 16.98)	3.993	<0.001
HOMA-IR		7.13 (5.14, 8.56)	5.31 (4.32, 6.50)	4.841	<0.001
HbA1c (%)		7.61 (6.83, 8.48)	7.54 (6.33, 8.99)	1.886	0.002
TG (mmol/L)		2.04 (1.56, 2.56)	1.96 (1.70, 2.23)	2.388	<0.001
TC (mmol/L)		3.97 (3.01, 4.82)	3.86 (3.28, 4.50)	1.615	0.011
HDL-C (mmol/L)		0.97 ± 0.16	1.01 ± 0.26	−2.916	0.004
LDL-C (mmol/L)		2.76 ± 0.43	2.67 ± 0.48	2.409	0.016
Diabetic progression	<10 yrs	79	355	226.132	<0.001
	≥10 yrs	166	42		
Family history of thyroid nodules	Yes	55	23	39.376	<0.001
	No	190	374		

Data are represented as counts, mean ± SD or median (IQR). BMI: Body mass index; HbA1c: Hemoglobin A1c; FBGL: Fasting plasma glucose; FINS: Fasting insulin; TG: Triglycerides; TC: Total cholesterol; LDL-C: Low-density lipoprotein cholesterol; HDL-C: High-density lipoprotein cholesterol.

### Ethical statement

Informed consent has been obtained from the guardians of all patients for publication.

### Statistical analysis

The data was analyzed using SPSS 27.0 statistical software. Measurement data with a normal distribution were expressed as mean ± SD, while non-normal distribution data were presented as median (interquartile range). The independent sample *t*-test was used to compare normal distribution measurement data, and the Kolmogorov–Smirnov test was used for non-normal distribution data. Categorical data were compared using a χ2 test, and logistic regression was used for multivariate analysis of different groups. A *P* value of less than 0.05 was considered statistically significant.

## Results

### Analysis of influencing factors of TNs in diabetic patients

This study included 642 patients with diabetes who underwent thyroid color ultrasonography, with 245 patients ultimately screened for TNs. Table 1 shows the analysis of risk factors for TNs in diabetic patients. Compared to controls, patients with TNs were significantly older (*P* < 0.001), but there was no significant difference in sex distribution (*P* > 0.05). In addition, BMI was significantly higher in patients with TNs than in controls (*P* < 0.001). The levels of FBGL, FINS, HOMA-IR, and HbA1c were significantly higher in patients with thyroid nodules compared to controls (all *P* < 0.01). In terms of lipid metabolism, the levels of TG, TC, and LDL-C in patients with TNs were significantly higher than those in the control group (all *P* < 0.05), while HDL-C levels were significantly lower than those in the control group (*P* < 0.01). Additionally, patients with diabetes for ten years or more had a significantly higher incidence of TNs (*P* < 0.001), and there was a higher proportion of patients with TNs who had a family history compared to controls (*P* < 0.001).

### Logistic regression analysis of influencing factors of TNs in diabetic patients

**Figure 1.**
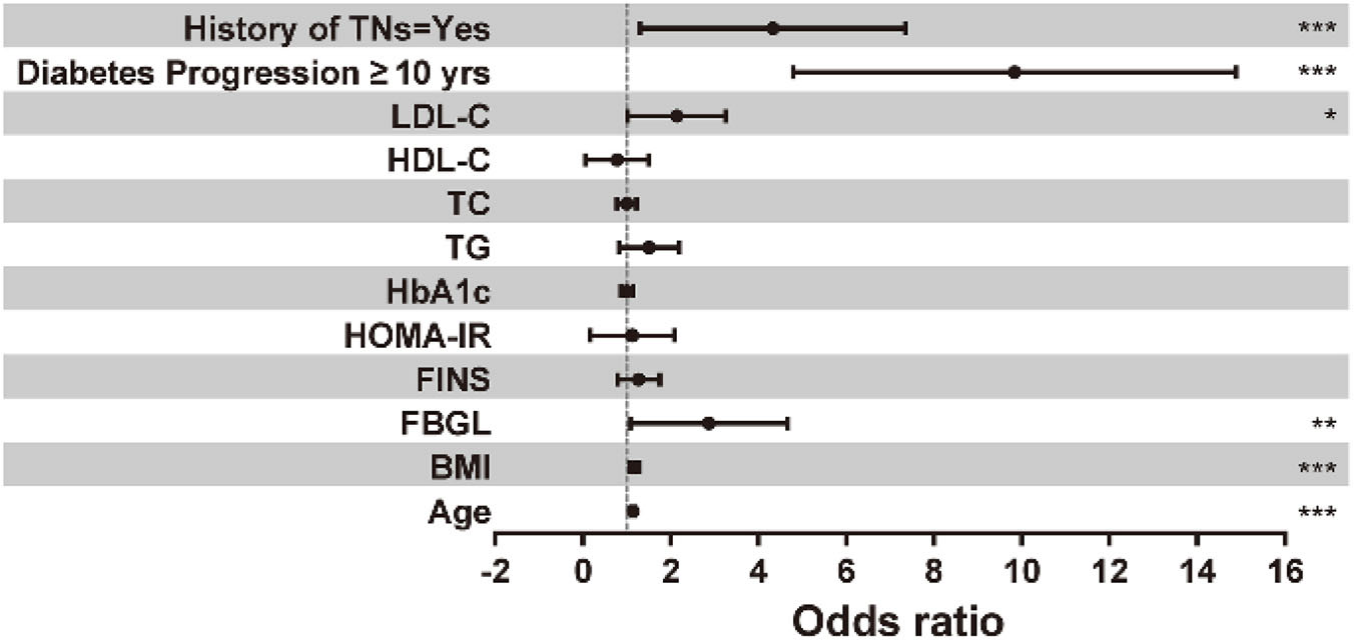
**Logistic regression analysis of influencing factors of thyroid nodules in diabetic patients.** TNs: Thyroid nodules.

The 12 candidate variables with *P* < 0.05 in the univariate were included in the multivariate logistic regression analysis and the results are shown in Table 2 and Figure 1. Age (OR ═ 1.149) and BMI (OR ═ 1.173) were independent risk factors for TNs in diabetic patients (*P* < 0.001). Specifically, for every 1-unit increase in age or BMI, there was a 14.9% and 17.3% increase in the risk of developing TNs in diabetic patients, respectively. While FINS, HOMA-IR, and HbA1c levels were included in the multivariate logistic regression analysis as metabolic indices of diabetes mellitus, their impact on the risk of TNs was not statistically significant (all *P* > 0.05). This indicates that these factors were not independent risk factors for the development of TNs. However, higher FBGL levels were identified as an independent risk factor (OR ═ 2.504, *P* < 0.01), with each 1-unit increase corresponding to a 1.504-fold increase in the risk of developing TNs in diabetic patients. Among the indices of lipid metabolism, an elevated LDL-C level was also found to be an independent risk factor for the development of TNs in diabetic patients (OR ═ 1.951, *P* < 0.05). For every 1-unit increase in LDL-C, there was a 95.1% increase in the risk of developing TNs. Additionally, having a diabetes course of ten years or longer or a family history of TNs were associated with 7.979 and 2.628 times higher likelihood, respectively, of developing TNs compared to diabetic patients with a diabetes course of less than ten years or no family history of TNs.

**Table 2:** Analysis of independent risk factors for thyroid nodules in diabetic patients

**Variable**	**B**	**SE**	**WaldX^2^**	***P***	**OR**	**95% CI**
Age	0.139	0.017	64.382	<0.001	1.149	(1.111–1.189)
BMI	0.160	0.048	11.143	<0.001	1.173	(1.068–1.289)
FBGL	0.918	0.335	7.527	0.006	2.504	(1.300–4.824)
FINS	0.196	0.196	1.006	0.316	1.217	(0.829–1.785)
HOMA-IR	−0.152	0.477	0.101	0.750	0.859	(0.337–2.189)
HbA1c	0.000	0.070	0.000	1.000	1.000	(0.871–1.148)
TG	0.343	0.238	2.083	0.149	1.410	(0.884–2.247)
TC	−0.018	0.116	0.024	0.876	0.982	(0.782–1.233)
HDL-C	−0.572	0.527	1.175	0.278	0.565	(0.201–1.587)
LDL-C	0.668	0.275	5.925	0.015	1.951	(1.139–3.342)
Diabetes progression ≥ 10 yrs	2.195	0.271	65.741	<0.001	8.979	(5.282–15.263)
Family history of thyroid nodules ═ Yes	1.289	0.380	11.522	<0.001	3.628	(1.724–7.635)

BMI: Body mass index; HbA1c: Hemoglobin A1c; FBGL: Fasting plasma glucose; FINS: Fasting insulin; TG: Triglycerides; TC: Total cholesterol; LDL-C: Low-density lipoprotein cholesterol; HDL-C: High-density lipoprotein cholesterol.

### Analysis of influencing factors of TNs with different ACR TR in diabetic patients

Further risk determination was performed for all patients with TNs by the ACR TI-RADS risk stratification system, and the influencing factors for ACR TI-RADS ═ 5 (TR5) nodules were analyzed. As shown in Table 3, there were no statistically significant differences in age, sex distribution, and BMI levels between TR5 and TR1–4 TNs (all *P* > 0.05). The levels of FBGL, FINS, HOMA-IR, and HbA1c in patients with TR5 TNs were significantly higher than those in patients with TR1–4 (all *P* < 0.05). In terms of lipid metabolism, LDL-C levels in patients with TR5 TNs were significantly higher than those in TR1–4 groups (*P* < 0.05). However, TG, TC, and HDL-C levels were not statistically different between patients with TR5 and TR1–4 TNs (all *P* > 0.05). Furthermore, patients with TR5 TNs had larger nodule diameters than those with TR1–4 nodules (*P* < 0.001). The proportion of patients with a family history of TNs was also significantly higher in the TR5 group compared to the TR1–4 group (*P* < 0.05).

**Table 3:** Univariate analysis of thyroid nodules by ACR TI-RADS classification in diabetic patients

**Variable**		**ACR TI-RADS ═ 1∼4(*n* ═ 211)**	**ACR TI-RADS ═ 5 (*n* ═ 34)**	**t/Z/X^2^**	***P***
Age (years)		56.00 (51.00, 61.00)	57.50 (50.00, 61.00)	0.536	0.936
Sex	Male	103	15	0.259	0.611
	Female	108	19		
BMI (kg/m^2^)		26.16 ± 2.42	27.03 ± 2.37	1.949	0.052
FBGL (mmol/L)		9.28 (8.41, 10.23)	9.81 (9.36, 10.37)	2.031	<0.001
FINS (mIU/L)		17.24 (11.80, 20.18)	19.51 (16.07, 22.72)	1.455	0.029
HOMA-IR		7.05 (5.09, 8.39)	8.44 (7.03, 9.59)	1.763	0.004
HbA1c (%)		7.53 (6.76, 8.37)	7.95 (7.53, 9.09)	1.450	0.030
TG (mmol/L)		2.00 (1.52, 2.56)	2.07 (1.67, 2.57)	0.728	0.664
TC (mmol/L)		3.89 (2.97, 4.71)	4.61 (3.30, 5.14)	1.300	0.068
HDL-C (mmol/L)		0.96 ± 0.16	0.99 ± 0.16	1.010	0.314
LDL-C (mmol/L)		2.73 ± 0.42	2.94 ± 0.42	2.692	0.008
Nodal diameter		4.58 (2.30, 6.46)	6.04 (4.76, 6.71)	2.000	<0.001
Diabetic progression	<10 yrs	96	11	2.057	0.152
	≥10 yrs	115	23		
Family history of thyroid nodules	Yes	42	13	5.651	0.017
	No	169	21		

Data are represented as counts, mean ± SD or median (IQR). ACR: American College of Radiology; FBGL: Fasting plasma glucose; FINS: Fasting insulin; BMI: Body mass index; HbA1c: Hemoglobin A1c; TG: Triglycerides; TC: Total cholesterol; HDL-C: High-density lipoprotein cholesterol; LDL-C: Low-density lipoprotein cholesterol.

**Table 4:** Analysis of independent risk factors of thyroid nodules by ACR TI-RADS classification in diabetic patients

**Variable**	**B**	**SE**	**Wald X^2^**	***P***	**OR**	**95% CI**
FBGL	−0.088	0.487	0.033	0.856	0.916	(0.353–2.376)
FINS	−0.149	0.260	0.330	0.566	0.861	(0.518–1.433)
HOMA-IR	0.590	0.580	1.033	0.309	1.803	(0.578–5.622)
HbA1c	0.448	0.182	6.084	0.014	1.566	(1.096–2.235)
LDL-C	0.969	0.531	3.336	0.068	2.637	(0.932–7.462)
Nodal diameter	0.360	0.108	11.049	0.001	1.433	(1.159–1.772)
Family history of thyroid nodules	1.004	0.460	4.765	0.029	2.729	(1.108–6.722)

ACR: American College of Radiology; FBGL: Fasting plasma glucose; FINS: Fasting insulin; LDL-C: Low-density lipoprotein cholesterol.

### Logistic regression analysis of influencing factors of TNs with different ACR TR in diabetic patients

**Figure 2.**
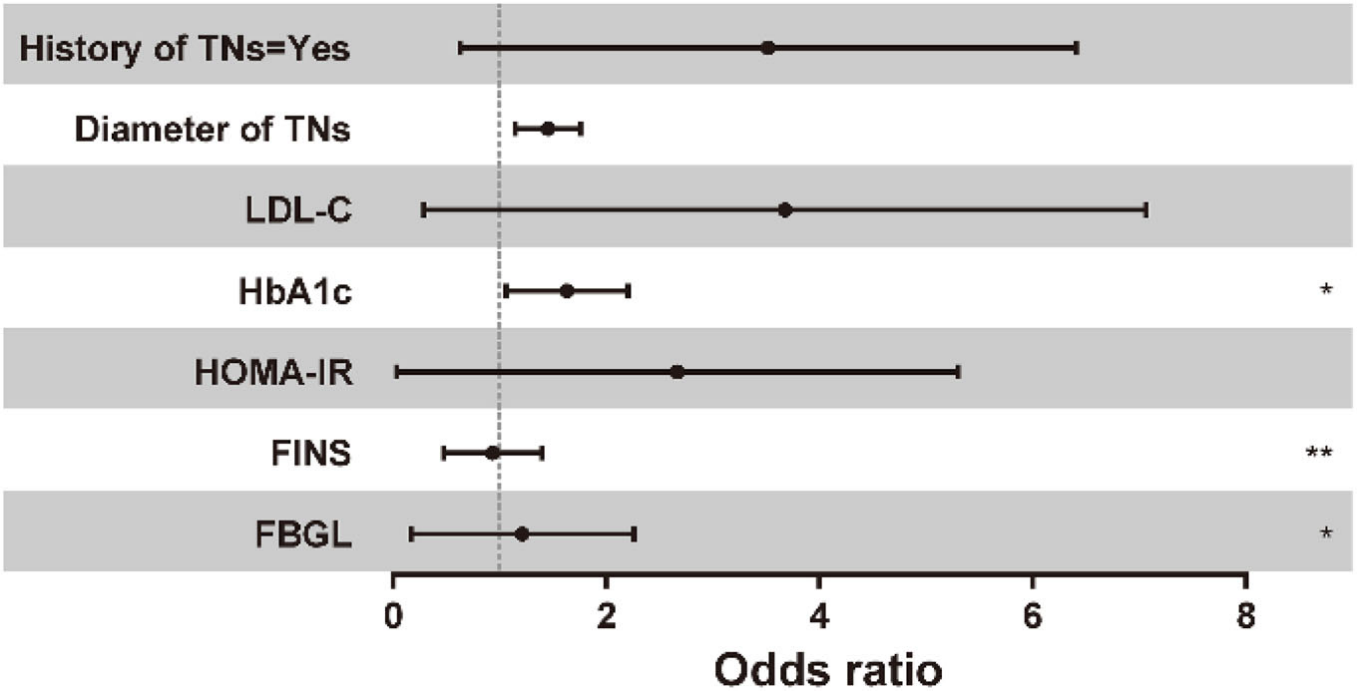
**Logistic regression analysis of influencing factors of thyroid nodules with different ACR TI-RADS classification**. ACR TI-RADS: American College of Radiology Thyroid Imaging Reporting and Data System.

Seven candidate variables with *P* < 0.05 in the univariate were included in the multivariate Logistic regression analysis and the results are shown in Table 4 and Figure 2. When FBGL, FINS, and HOMA-IR levels were included in multivariate logistic regression analysis, the significant effect on the risk of TR5 TNs disappeared (all *P* > 0.05), suggesting that these factors were not independent risk factors for the development of TR5 thyroid. In addition, HbA1c was an independent risk factor for the development of TR5 TNs (OR ═ 1.566, *P* < 0.05). When HbA1c was increased by 1, diabetic patients had an independent 56.6% increased risk of developing TR5 TNs. There was no significant independent effect of LDL-C on the occurrence of TR5 TNs (*P* > 0.05). In addition, the nodule diameter was an independent risk factor for the development of TR5 TNs (OR ═ 1.433, *P* < 0.01). This indicates that diabetic patients with a family history of TNs have a 1.729-fold increased risk compared to those without a family history.

### Consistency analysis of TNs with different ACR TR and Bethesda score in patients with diabetes mellitus

All 236 study participants underwent FNAC and results were analyzed concordantly using the ACR TI-RADS and Bethesda scoring systems. Table 5 displays the results for 211 patients. 28 nodules unable to be adequately classified as benign or malignant (Bethesda 1 ═ 4, Bethesda 3 ═ 10, Bethesda 4 ═ 11) were excluded from the analysis. Among the remaining nodules classified as TR3, only 2 had a Bethesda score of 5, while the others (*n* ═ 67) were classified as Bethesda score 2. Of these TR3 nodules, 97.10% (67/69) were pathologically confirmed as benign, demonstrating 97.10% agreement with the Bethesda scoring system. Out of the 112 TR4 nodules, 15 were classified as Bethesda score 5 and 9 as Bethesda score 6. Of these, 88 were confirmed as benign, indicating that 78.57% (88/112) did not support the ACR TI-RADS recommendation. However, it should be noted that five nodules with a Bethesda score of 5 showed negative pathological findings. For TR5 nodules, which were recommended for FNAC, 12 had a Bethesda score of 5 and 18 had a Bethesda score of 6. All TR5 nodules were confirmed as malignant, supporting the ACR TI-RADS recommendation.

**Table 5:** Consistency analysis of thyroid nodules and Bethesda scores by ACR TI-RADS classification

**Bethesda score**	**ACR TI-RADS classification**									
	**1**		**2**		**3**		**4**		**5**	
	**+**	**−**	**+**	**−**	**+**	**−**	**+**	**−**	**+**	**−**
2	0	2	0	4	0	67	0	88	0	0
5	0	0	0	0	2	0	10	5	12	0
6	0	0	0	0	0	0	9	0	18	0
*X^2^*	185.003									
*P*	<0.001									

ACR: American College of Radiology; ACR TI-RADS: American College of Radiology Thyroid Imaging Reporting and Data System.

## Discussion

The thyroid gland is one of the target tissues that is affected by metabolic disorders [19]. Among endocrine and metabolic system issues, T2DM and thyroid disease stand out as the most prevalent [20]. Diabetes mellitus has been shown to be closely related to the development of TNs, and there is a positive correlation between blood glucose and the formation of TNs [21]. IR, characterized by hyperglycemia and high insulin levels, serves as the primary trigger in the onset of most type 2 diabetes cases. Excessive insulin can bind to insulin-like growth factor binding proteins, thereby increasing levels of free insulin-like growth factor 1 (IGF-1) in the blood [22]. IGF-1 and its receptors have been shown to be expressed in follicular and C cells of the thyroid gland and involved in cell regulation and proliferation [23]. One of the target tissues for the disturbance of glucose metabolism is the thyroid gland, and the statement that IR and hyperinsulinemia are associated with the development of TNs has been confirmed by Ayturk et al. [24]. In addition, Auchincloss et al. [25] reported that insulin receptors are expressed in both thyroid cells and insulin and that increased insulin levels may reduce production of IGF-1 binding proteins, leading to elevated levels of free IGF-1, which subsequently stimulates protein and DNA synthesis and promotes mitosis in thyroid cells. Belfiore [26] found that insulin itself is a pro-cytokine that can induce the growth, differentiation, and proliferation of thyroid cells and stimulate TN formation. In the present study, FBGL, FINS, HOMA-IR, HbA1c levels, and diabetes progression were significantly higher in patients with T2DM and TNs than in the non-nodular group. In addition, the results of logistic regression analysis showed that both FBGL level and diabetes progression were independent risk factors for the development of TNs. Currently, the exact mechanism linking disturbances in glucose metabolism to TNs remains unclear. However, we hypothesize that insulin not only acts as a glucose-lowering hormone but also promotes vascular endothelial cell proliferation. Prolonged exposure to high glucose levels in poorly controlled type 2 diabetes patients can lead to IR and mild inflammatory responses in cells and tissues, thereby promoting angiogenesis in TNs.

Currently, due to the widespread use of high-frequency ultrasound in TN diagnosis, there has been a significant increase in nodule detection rates. The ACR TI-RADS grading system serves as a method for assessing the risk of thyroid malignancy based on ultrasonographic features. It is generally accepted that TR1–3 nodules can be considered benign nodules, while TR4–5 should be highly suspected of malignancy [27]. However, recent studies have revealed that the optimal diagnostic threshold on the ROC curve for distinguishing between benign and malignant TNs is > TR4, indicating that TR5 nodules are highly suspicious for malignancy. Therefore, we categorized all patients with TNs into TR1–4 and TR5 groups to further explore the factors influencing the malignancy of TNs. The results of this study showed that FBGL, FINS, HOMA-IR, and HbA1c levels were significantly higher in patients with TR5 TNs than in patients with TR1–4. Furthermore, logistic regression analysis identified HbA1c levels as an independent risk factor for elevated TN grade in patients with T2DM. This may be attributed to impaired glucose tolerance leading to IR, subsequently triggering a chronic inflammatory response and increasing TN grade. Additionally, Yildirim et al. [28] discovered that IR poses a risk factor for papillary thyroid cancer, indicating a significant link between IR and malignant thyroid lesions, which aligns with our findings.

In this study, no statistically significant difference was observed in the distribution of sexes between TNs and controls, nor between the different ACR TI-RADS groups. This finding contrasts with the research by Xu et al., which suggested a higher likelihood of TNs in women [29]. This inconsistency could be attributed to the relatively small sample size of our study and the specific selection of diabetic patients. It is known that cellular hyperplasia and fibrosis in the thyroid gland increase with age, contributing to the formation of TNs [6]. However, it has also been suggested that the increased incidence of TNs in the elderly is largely attributable to more extensive thyroid ultrasonography in the elderly population [30]. The results of this study indicate that age independently acts as a risk factor for TNs in diabetic patients, with the risk increasing by 14.9% for each additional year of age. This finding is in line with Guth et al.’s [30] research, which demonstrated a higher prevalence of TNs among the elderly, with nearly 80% incidence in individuals aged ≥ 60 years. However, it has been shown that the incidence of malignant TNs decreases with age [31]. A study of thyroid fine-needle aspiration revealed that the prevalence of thyroid cancer was 17% to 23% in the 20–49-year-old group, compared to only 13% in patients aged 70 years (±14 years), suggesting a reduced prevalence of malignant nodules in older patients [31]. Interestingly, our study found no statistically significant difference in age between patients with TR5 and those with TR1–4 TNs, suggesting that age may not play a significant role in the degree of thyroid malignancy.

In 2015, Bétry et al. found that individuals with higher BMI can stimulate abnormal proliferation and differentiation of individual thyroid tissues by inducing local systemic metabolic disturbances, causing abnormalities in the hypothalamic–pituitary thyroid axis. Similarly, Sari et al. [32] discovered that higher BMI and body fat content were associated with elevated concentrations of thyroid-stimulating hormone (TSH) and larger thyroid gland volumes. Similarly, Kitahara et al. [33] discovered that higher BMI and body fat content were associated with elevated concentrations of TSH and larger thyroid gland volumes. In addition, Kitahara et al. conducted a prospective study of 434,953 men and 413,3979 women in the United States and demonstrated a significant positive association between BMI and thyroid cancer risk. However, in our study, BMI levels did not differ statistically between TR5 and TR1–4 nodules, possibly due to the small sample size. Furthermore, our study demonstrated that levels of TG, TC, and LDL-C were notably higher in diabetic patients with TNs compared to controls, with LDL-C levels being identified as independent risk factors for TN development in diabetic patients. Lee and Wang [34] have shown a close relationship between TC, LDL-C, and TSH levels in patients with hyperthyroidism, suggesting that lipid metabolism influences TSH levels, thereby influencing TNs. The current “hypothalamic–pituitary–thyroid–adipose tissue balance” hypothesis may explain the mechanism by which lipids affect TNs [34]. Elevated blood lipids, which can induce leptin resistance, play a role in regulating the expression of thyrotropin-releasing hormone genes [34]. Clinically, dyslipidemia usually coexists with thyroid disorders, and both hypersecretion and hypothyroid hormone can cause dyslipidemia [35]. Hypersecretion of thyroid hormones enhances metabolism, leading to decreased lipid levels, while decreased thyroid hormone secretion slows metabolism, resulting in elevated lipid levels. Additionally, excessive fat deposition in individuals with hyperlipidemia can increase the autoinflammatory response and promote the overexpression of inflammatory factors, such as IL-6 and MCP-1, potentially contributing to TN formation [36].

At present, the treatment of TNs primarily falls into two categories. Benign nodules are typically managed through observation, with surgery or radiofrequency ablation performed electively if compression symptoms arise [37]. Conversely, if nodules exhibit malignant characteristics, surgical intervention is usually warranted [37]. Therefore, distinguishing between benign and malignant TNs remains a pivotal clinical concern [38]. At present, FNAC is the most effective method of differentiating between benign and malignant TNs [39]. However, given that the majority of nodules are benign, and FNAC is an invasive procedure, not all nodules necessitate FNAC [40]. Hence, there is a need for an effective, noninvasive approach to identify which nodules require FNAC. ACR TI-RADS offers a set of simple, easy, and standardized classification criteria, effectively mitigating diagnostic bias arising from varying levels of ultrasound expertise among practitioners. Hoang et al. [41] utilized ACR TR to assess the diagnostic accuracy of TNs and found notable improvement in diagnostic precision. The results of this study show that 97.10% of TR3 nodules in diabetic patients with TNs do not recommend further FNAC examination as their pathological findings were benign, contradicting the ACR TI-RADS recommendation of FNAC for TR3–TR5 nodules. Additionally, nodule diameter emerged as an independent risk factor for the development of TR5 nodules (OR ═ 1.433, *P* < 0.01). Consequently, nodal malignancy risk increased by 86.6% when the nodule diameter reached 3 cm. Thus, we propose that TR3 nodules ≥ 3 cm in diameter should be considered for further FNAC examination.

Furthermore, among 112 TR4 nodules, 88 were benign, indicating that 78.57% (88/112) of these nodules did not align with the ACR TI-RADS recommendation. Hence, we suggest that the determination of TR4 nodules for further FNAC examination should be based on additional parameters, such as nodule size, morphology, calcification, etc. Moreover, all TR5 nodules were malignant, affirming the ACR TI-RADS recommendation.

There are several limitations to this study: (1) It relies on a single data source, lacks a multicenter control analysis, and the sample size is small, potentially introducing bias and limiting the generalizability of the results to other clinical settings and patient groups; (2) Due to space constraints, factors, such as patient lifestyle, iodine status, occupational exposure, nodule characteristics, and other related environmental factors were not included in the analysis; and (3) The distinction between benign and malignant subgroups within TR4 nodules remains unexplored and warrants further investigation.

## Conclusion

In summary, TNs in patients with T2DM are linked to age, BMI, and metabolic factors like blood sugar and lipid levels. The risk of TN malignancy is also associated with blood sugar and lipid metabolism indicators. Patients with T2DM generally exhibit a low risk of malignancy within the TR3 classification of TNs, thus further FNAC testing is typically not recommended. However, nodules classified as TR3 with a diameter of 3 cm or more may need further FNAC assessment. For nodules classified as TR4–TR5, additional FNAC should be considered on an individual basis. When using the ACR TI-RADS risk assessment system, it may be necessary to clarify the nodule category, scoring criteria, etc., to accurately diagnose and distinguish between benign and malignant nodules, and guide clinical management more effectively.

## Data Availability

Data sharing is not applicable to this article as no datasets were generated or analyzed during the current study.
